# Retention of Key Characteristics of Unprocessed Chorion Tissue Resulting in a Robust Scaffold to Support Wound Healing

**DOI:** 10.3390/ijms242115786

**Published:** 2023-10-31

**Authors:** Katrina A. Harmon, MaryRose Kammer, Justin T. Avery, Kelly A. Kimmerling, Katie C. Mowry

**Affiliations:** Organogenesis, Birmingham, AL 35243, USA

**Keywords:** hypothermic storage, placental membranes, chorion, scaffold

## Abstract

Placental membranes have been widely studied and used clinically for wound care applications, but there is limited published information on the benefits of using the chorion membrane. The chorion membrane represents a promising source of placental-derived tissue to support wound healing, with its native composition of extracellular matrix (ECM) proteins and key regulatory proteins. This study examined the impact of hypothermic storage on the structure of chorion membrane, ECM content, and response to degradation in vitro. Hypothermically stored chorion membrane (HSCM) was further characterized for its proteomic content, and for its functionality as a scaffold for cell attachment and proliferation in vitro. HSCM retained the native ECM structure, composition, and integrity of native unprocessed chorion membrane and showed no differences in response to degradation in an in vitro wound model. HSCM retained key regulatory proteins previously shown to be present in placental membranes and promoted the attachment and proliferation of fibroblasts in vitro. These data support the fact that hypothermic storage does not significantly impact the structure and characteristics of the chorion membrane compared to unprocessed tissue or its functionality as a scaffold to support tissue growth.

## 1. Introduction

Human placental membranes have a long history of use in a variety of wound applications, with their first use documented in the early 1900s [1,2,3]. These membranes have also been adapted for use in surgical repair and reconstructive surgeries, oral surgeries, and ocular reconstructions [4,5,6,7]. Several clinical studies have demonstrated the benefit of placental membranes to support wound healing [8,9,10,11,12], while pre-clinical studies have shown placental membranes to be a source of growth factors and cytokines that provide an extracellular matrix (ECM) scaffold for cell attachment and growth [13,14,15,16].

While placental membranes as a whole have been widely studied and used clinically, there are limited studies evaluating the potential benefits of the chorion membrane independently. The chorion membrane, comprising the reticular, basement membrane, and trophoblast layers, is thicker than the amnion membrane with a robust ECM and has been reported to be a significant contributor of growth factors and cytokines, including tissue inhibitors of metalloproteases (TIMPs), which inhibit matrix metalloproteases (MMPs) [15,17,18]. While the balance of MMPs and TIMPs is known to be important for regulating collagen deposition, aberrant or prolonged expression of MMPs is known to be a major factor in non-healing and chronic wounds [19,20]. In vitro, chorion membranes have been shown to serve as a scaffold for various cell types [16,21], and clinically, chorion membrane has been shown to significantly improve the healing of surgical and chronic wounds [9,22]. The characteristics of chorion membranes present a promising treatment modality to support tissue remodeling during the wound healing cascade.

A hypothermically stored chorion membrane (HSCM) was recently developed using a proprietary process, which aims to maintain native tissue attributes, including ECM structure, composition, key regulatory proteins, and viable cells. Maintaining a hydrated state through processing has been reported to result in better cellular attachment and infiltration than dehydration, likely due to maintaining the structural and biological characteristics of the ECM [23]. In the current study, we evaluated the impact of hypothermic processing on chorion membranes and compared them to donor-matched unprocessed and dehydrated tissue. Tissue structure, composition, and ECM integrity were assessed in addition to degradation characteristics using an in vitro simulated wound fluid (SWF) model. HSCM was further characterized for proteomic content and functionality as a scaffold for cell growth to support the wound healing environment.

## 2. Results

### 2.1. Hypothermic Storage Maintains Native Placental Membrane Tissue Structure and Composition

**Figure 1:**
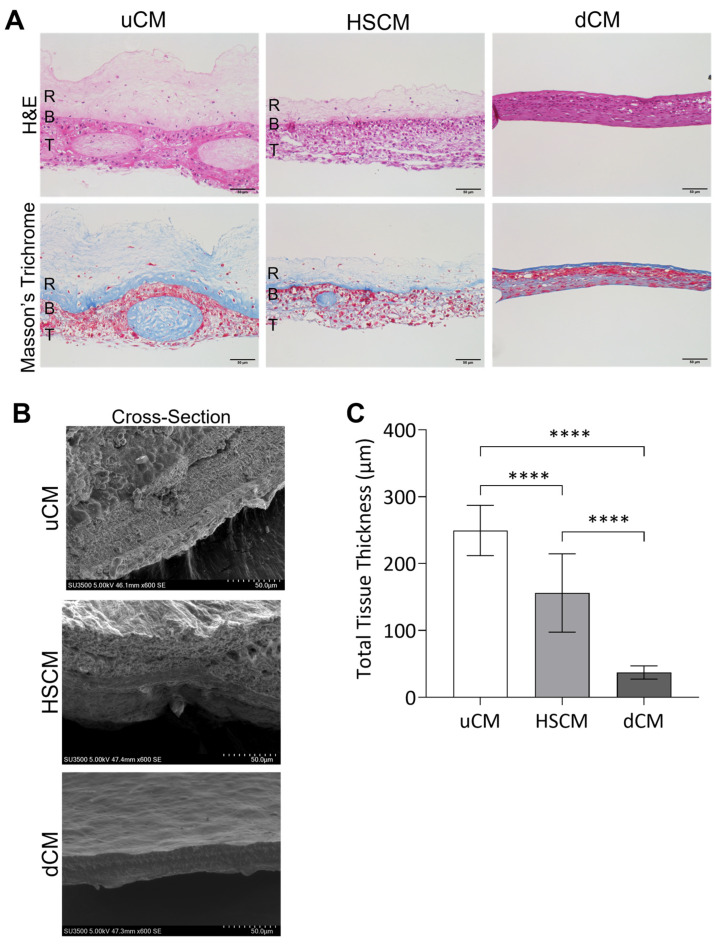
Structural characterization of chorion membranes. (**A**) Hematoxylin and eosin (H&E) and Masson’s trichrome stains and (**B**) scanning electron microscopy of uCM, HSCM, and dCM. (**C**) Total thickness measurements. Average ± standard deviation reported (*n* = 8 measurements per donor; 3 donors total). **** *p* ≤ 0.0001. R = reticular layer; B = basement membrane; T = trophoblast layer; uCM = unprocessed chorion membrane; HSCM = hypothermically stored chorion membrane; dCM = dehydrated chorion membrane. SEM = 600× magnification. H&E and Masson’s trichrome = 20× magnification. A 50 µm scale bar is used for all images.

Histological staining was utilized to characterize unprocessed, hypothermically stored, and dehydrated chorion membranes (Figure 1A). Unprocessed chorion membrane (uCM) showed well-defined reticular, basement membrane, and trophoblastic layers, with an open matrix structure. Hypothermically stored chorion membranes (HSCMs) were consistent with uCM, maintaining the membrane layers, porosity, and open matrix structure, though a decrease in thickness was observed. Dehydrated chorion membranes (dCM) resulted in graft compression, loss of porosity, and ECM layers that were not easily identifiable compared to uCM and HSCM. While both uCM and HSCM maintained a lightly colored, open ECM, dCM resulted in darkly stained and dense ECM. Tissue structure was further characterized via scanning electron microscopy (SEM) images of uCM, HSCM, and dCM (Figure 1B). All membranes presented with no observable sidedness and cross-sectional views of unprocessed and hypothermically stored membranes revealed discrete ECM layers. By measuring the thickness of uCM, HSCM, and dCM, it was found that dCM was 85% thinner than uCM and 76% thinner than HSCM (Figure 1C). HSCM was 47% thinner than uCM, likely due to the debridement step that takes part during the processing of HSCM. uCM was significantly thicker than both HSCM (*p* = 0.0001) and dCM (*p* = 0.0001), while HSCM was significantly thicker than dCM (*p* = 0.0001). Due to the significant structural changes and ECM compression in dCM compared to uCM and HSCM, we continued the study to assess the maintenance of unprocessed characteristics with HSCM compared to uCM.

**Figure 2:**
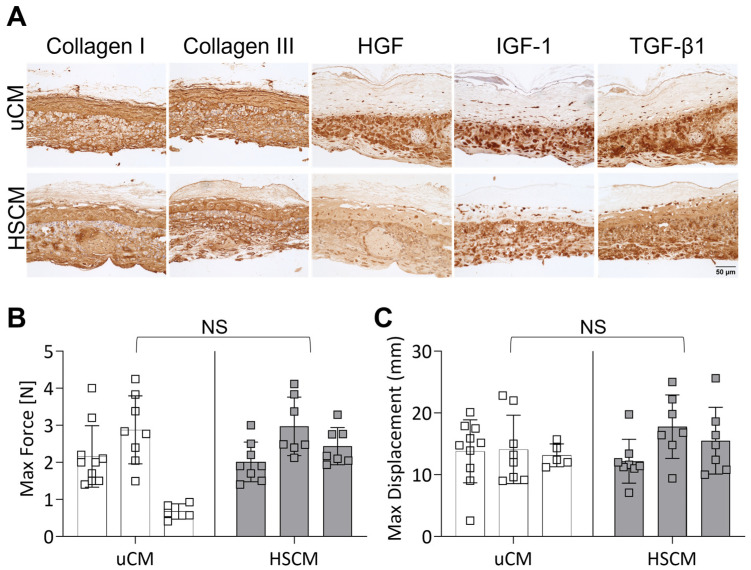
Histological evaluation and mechanical testing of unprocessed and hypothermically stored chorion membranes. (**A**) Immunohistochemistry of relevant ECM proteins and cytokines and growth factors on uCM and HSCM. (**B**) Max force and (**C**) max displacement of chorion membranes. uCM = unprocessed chorion membrane; HSCM = hypothermically stored chorion membrane; HGF = hepatocyte growth factor; IGF-1 = insulin-like growth factor 1; TGF-β1 = transforming growth factor-beta 1. In all images, 20× magnification and a 50 µm scale bar are used. Average ± standard deviation reported (*n* = 5–10 measurements per donor; 3 donors total). NS denotes not significant.

To further evaluate the maintenance of unprocessed characteristics, HSCM and uCM were subjected to immunohistochemical (IHC) staining and assessed for the presence and distribution of ECM proteins, as well as key growth factors and cytokines known to be present in placental membranes (Figure 2A). The ECM proteins collagens I and III were found to be distributed throughout uCM and HSCM and were highly concentrated in the basement membrane and trophoblastic layers. Transforming growth factor beta 1 (TGF-β1) and insulin-like growth factor 1 (IGF-1) were present in chorion tissues, and the expression was similar between uCM and HSCM. Interestingly, small differences in hepatocyte growth factor (HGF) expression were observed, with uCM presenting slightly stronger expression.

The impact of hypothermic preservation on the ECM structure and functionality was evaluated further by tensile testing and presented as maximum force and displacement. In response to the hypothermic storage of chorionic membranes, there was no significant difference in either maximum force (Figure 2B; *p* = 0.4749) or displacement (Figure 2C; *p* = 0.4714) compared to unprocessed chorion. Though not significant, inter- and intra-donor variability was seen.

### 2.2. Maintenance of Degradation Profile of Chorion Membrane In Vitro

**Figure 3:**
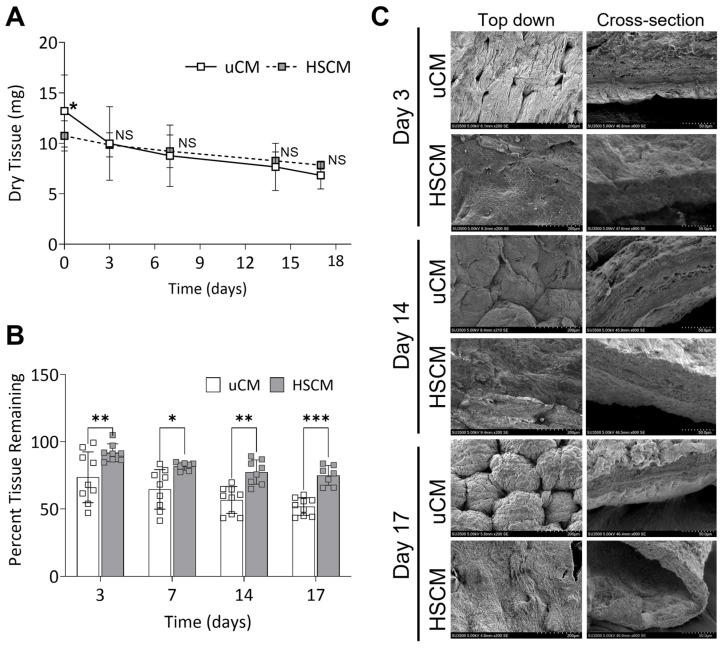
Chorion membranes after degradation in an in vitro simulated wound fluid model. (**A**) Dry tissue remaining and (**B**) percent tissue remaining after 17 days of in vitro degradation. (**C**) Representative scanning electron images of degraded chorion membranes. uCM = unprocessed chorion membrane; HSCM = hypothermically stored chorion membrane. Average ± standard deviation reported (*n* = 3 per donor; 3 donors total). * *p* ≤ 0.05, ** *p* ≤ 0.01, *** *p* ≤ 0.001, NS denotes not significant. In all images, 200× magnification and a 200 µm scale bar are used.

Similarities in the responses of uCM and HSCM following in vitro degradation in simulated wound fluid (SWF) for up to 17 days were evaluated. Following culture in SWF, though some degradation was observed, both uCM and HSCM withstood rapid degradation and there were no significant differences (*p* = 0.1451) between their slopes (Figure 3A). Initial differences in tissue weight (*p* = 0.0308) were observed at day 0 as expected due to the processing methodology for HSCM (Figure 3A). There were no significant differences in tissue weight at any other timepoints tested. However, HSCM resulted in significantly greater percent tissue remaining after 3 (*p* = 0.0046), 7 (*p* = 0.0115), 14 (*p* = 0.0011), and 17 (*p* = 0.0004) days of in vitro degradation compared to uCM (Figure 3B). SEM images revealed that as uCM degraded over 17 days, pockets within the membrane began to develop, deepen, and change in appearance (Figure 3C). Similarly, pockets present on HSCM also began to deepen and by day 14, the surface began to flatten, losing discernable definition. Cross-sectional analysis of uCM and HSCM revealed gaps within the matrix and increased layer separation over time (Figure 3C).

### 2.3. Chorion Membrane Retains Growth Factors and Cytokines

**Figure 4:**
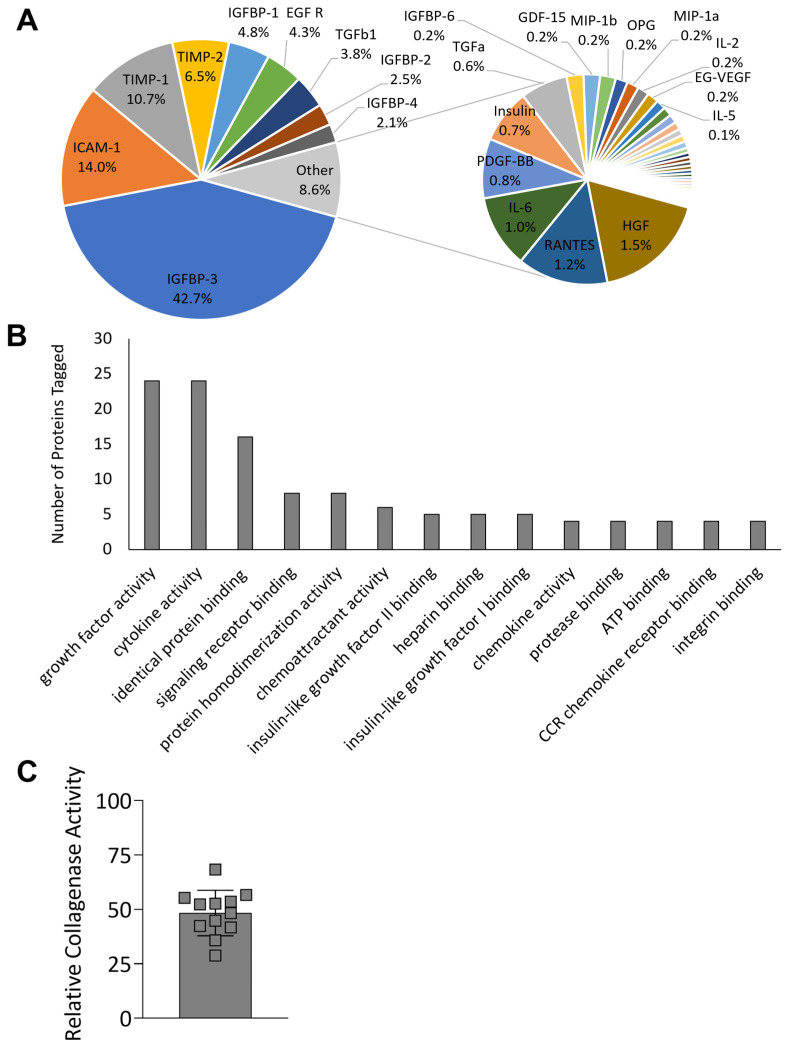
Proteomic characterization and collagenase inhibition of hypothermically stored chorion membrane. (**A**) Relative protein content of 53 proteins detected at greater than 10 pg/mL and (**B**) most abundant molecular functions of proteins detected. (**C**) Collagenase inhibition activity compared to non-inhibited control. uCM = unprocessed chorion membrane; HSCM = hypothermically stored chorion membrane. Average ± standard deviation reported (*n* = 3 per donor, 3 donors total for proteomics; *n* = 3–6 per donor, 3 donors total for collagenase inhibition).

The proteomic content of HSCM was evaluated to determine the retention of key growth factors and cytokines. uCM was not included for further analysis, as HSCM was shown to maintain the key characteristics of unprocessed tissue following storage. The hypothermic storage of chorion membrane retained 53 targets, with concentrations greater than 10 pg/mL (Figure 4A). Insulin-like growth factor binding protein 3 (IGFBP-3), intercellular adhesion molecule 1 (ICAM-1), and TIMPs were some of the most prevalent targets detected, with TIMP-1 contributing 11% and TIMP-2 7% of the total proteins detected. The most abundantly tagged molecular functions, describing four or more proteins each, were graphed (Figure 4B), and included growth factor activity (24 proteins), cytokine activity (24 proteins), chemoattractant activity (6 proteins), and protease binding (4 proteins). Complete results are included in Supplementary Table S1. Of particular interest due to the high level of TIMPs found in the chorion, the functionality of HSCM was further evaluated using an in vitro MMP inhibition assay. HSCM resulted in a 52% reduction in collagenase IV activity (Figure 4C). 

### 2.4. Hypothermically Stored Chorion Membrane Supports Cell Attachment In Vitro

**Figure 5:**
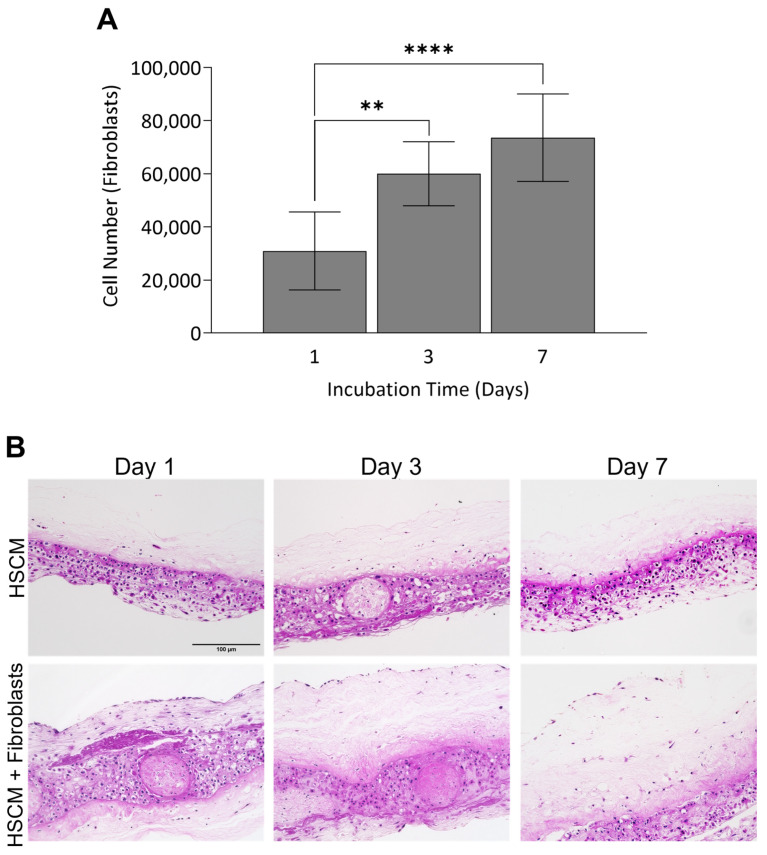
Fibroblast attachment and growth on hypothermically stored chorion membrane. (**A**) Fibroblast cell attachment on hypothermically stored chorion membrane out to 7 days in culture as measured by AlamarBlue and (**B**) representative H&E images of control (non-seeded) and seeded grafts. HSCM = hypothermically stored chorion membrane. Average ± standard deviation reported (*n* = 3 per cell lot; 2 cell lots total). ** *p* ≤ 0.01, **** *p* ≤ 0.0001. In all images, 200× magnification and a 150 µm scale bar are used.

Having demonstrated the maintenance of the structure and function of the ECM, as well as the retention of key regulatory proteins in HSCM, the utility of HSCM to serve as a scaffold for cell attachment and proliferation was evaluated in vitro using primary human fibroblasts. Primary fibroblasts robustly attached to HSCM and proliferated out to day 7, with significant increases in cell number between day 1 and day 3 (*p* = 0.0032) and 7 (*p* ≤ 0.0001) (Figure 5A). At each timepoint, to visually assess cell infiltration, H&E staining was performed on cross-sections of HSCM (Figure 5B). Cellular staining throughout the tissue indicates that cells not only attached to and proliferated on, but also infiltrated into the HSCM. Together, these data support the fact that HSCM serves as a robust scaffold to support cell attachment in vitro.

## 3. Discussion

While chorion membrane has not been widely studied, it has been shown to support wound healing in surgical and chronic wounds [9,24]. In this study, the impact of two preservation methods, hypothermic storage and dehydration, were evaluated for changes in composition and structural integrity and compared to unprocessed chorion. The results demonstrate that the hypothermic storage of chorion membranes retained the distinct layers and open structure of unprocessed tissue. Hypothermic storage resulted in a significant decrease in matrix thickness; however, this is expected as a result of processing. Dehydration and loss of water content in dCM resulted in an altered ECM structure and a substantial decrease in matrix thickness, and it been previously reported that dehydration results in a compromised ECM structure and a substantial loss in growth factor and cytokine concentrations [14,25]. Ultrastructural changes as a result of dehydration may result in the formation of physical bonds between collapsed ECM molecules, potentially altering degradation characteristics and the potential for cellular invasion [23]. Because of these factors, dCM was not carried forward for further assessment. 

ECM proteins in their normal biological structures within intact tissues are known to stimulate and regulate cellular proliferation, migration, and differentiation through a number of mechanisms; therefore, the maintenance of the native ECM within a biological tissue is expected to exhibit greater responses than those whose ECM has been disrupted [23,26]. HSCM maintained ECM and key regulatory proteins that are characteristically present in unprocessed chorion membranes [17]. The distribution of ECM proteins and growth factors throughout HSCM was comparable to donor-matched unprocessed tissues. TGF-β1, which was present in both uCM and HSCM, has a known role in tissue remodeling, specifically playing a role in collagen type I and III production and inhibiting MMP-1, -3, and -9 [27]. Mechanical testing revealed that chorion membranes are elastic, which is consistent with other studies [28,29]. In this study, there were no significant mechanical differences between unprocessed and hypothermically stored chorion membranes. As expected, intra- and inter-donor variability in mechanical properties was seen, which is expected due to differences associated with human donor tissues. 

An important assessment used to evaluate ECM functionality and the maintenance of unprocessed characteristics is speed and pathways of degradation. In this study, an in vitro model was developed to simulate the wound environment. The results demonstrate that HSCM not only remained resistant to rapid degradation, but also had comparable rates of degradation to uCM. While this has not been previously studied for chorion membranes, the results are consistent with previous studies, which reported that placental membranes are degraded after approximately four weeks [30]. While the SWF model used in this study has been previously published as a simulated wound model [31], the persistent inflammation associated with chronic wounds would likely result in accelerated degradation. In a clinical study, hypothermically stored amniotic membrane (HSAM) persistence was evaluated during the treatment of venous leg ulcers, and in this environment, HSAM was almost absent after seven days following treatment [32]. 

After confirming the similarities in the structure, ECM content, and durability of HSCM to unprocessed chorion membrane, HSCM proteomic content was assessed. Chorion has been previously shown to contain a variety of growth factors and cytokines [13,15], many of which were detected in HSCM. Specifically, IGFBP-3 was present in HSCM and encompassed over 40% of the overall protein content. IGFBP-3 serves an important role in IGF-I binding, resulting in the increased persistence of IGF-I, which has been shown to promote fibroblast proliferation and wound epithelialization in vivo and in vitro [33,34]. TIMP-1 and -2 made up over 15% of the proteins present in HSCM, and functionally, HSCM was shown to inhibit collagenase activity in vitro. Collagenases are enzymes from the MMP family (MMP-1, -8, -13) that are naturally inhibited by TIMP-1 and -2 [35,36]; Lobmann reported MMP-1 and -8 expression was sixty-five fold and two-fold higher, respectively, in biopsies taken from diabetic foot ulcers compared to acute traumatic wounds in non-diabetic patients, highlighting the importance of TIMPs in the inhibition of collagenases [37].

HSCM scaffolds are representative of unprocessed chorion membrane, retaining key regulatory proteins, resisting rapid degradation, and acting as a scaffold for cellular growth and invasion. This study has limitations, including the fact that the characterization of proteins in HSCM was limited to the evaluation of 80 cytokines related to inflammation and growth factors, while placental membranes have previously been shown to contain at least 640 proteins and growth factors [38]. Additionally, the proximity of maternal decidua to the trophoblast layer of the chorion membrane may raise concerns around immunogenicity; however, specific processing steps including the debridement of the outer layer of the chorion were incorporated to minimize any potential risks. Objective evidence of this removal is apparent in the 47% reduction in thickness post processing of HSCM compared to unprocessed chorion. Lastly, this work was limited to in vitro studies, whereas clinically, there are complex interactions between a multitude of cell types, enzymes, proteases, and inflammation that may play a role in the functionality of HSCM and any other graft.

Based on the data presented here, the hypothermic processing and storage of chorion (HSCM) maintains the key characteristics and functions of unprocessed chorion tissue, potentially delivering a promising treatment for wound care. Coupled with its durability, the treatment of wounds with HSCM may help reduce the number of applications needed to support the healing of challenging wounds. Future clinical studies are needed to validate the beneficial characteristics of chorion membrane to support progression through the phases of wound healing.

## 4. Materials and Methods

### 4.1. Human Tissue Allograft

Placental tissue was donated following informed consent after planned cesarean section, and all processing was completed in accordance with the Food and Drug Administration’s Good Tissue practices and the American Association of Tissue Banks’ standards. All tissue donations underwent medical social history screening and were tested for infectious diseases, including immunodeficiency virus, human T-lymphotropic virus I/II, hepatitis B and C, and syphilis. The chorion, from three independent donors, was separated from the amnion and was further processed in one of three ways: (1) maintained unprocessed, (2) dehydrated, or (3) hypothermically stored. Unprocessed chorion membranes (uCM) were utilized within 24 h of processing, while dehydrated chorion membranes (dCM) were dried overnight prior to use in assays outlined below. Hypothermically stored chorion membrane (HSCM; Novachor^®^, Organogenesis, Canton, MA, USA) was aseptically processed using a proprietary process, Allofresh™, and stored for up to 42 days until use within experiments. Of note, HSCM processing includes a debridement step that removes the outer portion of the trophoblast layer.

### 4.2. Tissue Characterization, Histological Assessment, and Tissue Thickness

Histological analysis was used to evaluate the impact of processing on the overall structure and composition of the unprocessed and processed tissues. Unprocessed, dehydrated, and hypothermically stored chorion membranes were fixed in 4% paraformaldehyde and embedded in paraffin. Serial sections 5 µm thick were cut from the tissue blocks and placed onto charged glass slides and dried overnight. Sections were then stained with histologic stains including hematoxylin and eosin (H&E) and Masson’s Trichrome Blue using standard techniques. Images were captured on an inverted microscope (Nikon Eclipse Ti, Nikon, Melville, NY, USA; EVOS M5000, ThermoFisher, Waltham, MA, USA).

To quantify tissue thickness, stitched H&E 10× images were divided into equal grids, numbered, and randomly selected using a sample size calculated based on a confidence level of 95%, margin of error of 20%, population proportion of 50%, and population size of 20. Tissue thickness was quantified using ImageJ (NIH, Bethesda, MD, USA). The values for each grid were averaged and used to calculate the tissue thickness per slide.

Tissue structure was further characterized via scanning electron microscopy (SEM). Membranes were dehydrated in an ethanol gradient to 100% and were critical point dried in a Denton DCP-1 Critical Point Dryer (Denton Vacuum, Moorestown, NJ, USA) per manufacturer’s instructions. Dried samples were mounted on aluminum stubs and coated with gold palladium using the Hummer 6.6 Sputter Coater (Anatech USA, Sparks, NV, USA) in a saturated argon environment. Images were obtained using a Hitachi SU3500 Scanning Electron Microscope (Hitachi High-Tech America Inc, Schaumburg, IL, USA) at 5 kV.

### 4.3. Immunohistochemistry

Immunohistochemical (IHC) staining was utilized to determine the presence and distribution of ECM proteins, growth factors, and cytokines within chorion tissue. Sections were deparaffinized and hydrated via gradient concentrations of ethanol to water. Antigen retrieval for samples stained for collagen I, collagen III, hepatocyte growth factor (HGF), and transforming growth factor-beta 1 (TGF-β1) (all antibodies; ThermoFisher, Waltham, MA, USA) was performed by placing slides into a buffer containing proteinase K (1:50 dilution) in Tris ethylenediaminetetraacetic acid (EDTA)-CaCl_2_ buffer at pH 8.0 for 15 min at 37 °C. Antigen retrieval for samples stained for insulin-like growth factor-1 (IGF-1; ThermoFisher, Waltham, MA, USA) was performed by placing slides into a buffer containing 0.01 M Tris + 1 mM EDTA at pH 9.0 for 20 min at 70 °C. After both antigen retrieval procedures were completed, slides were washed with deionized water and transferred to a 0.05 M Tris-based solution in 0.15 M NaCl with 0.1% *v*/*v* Triton-X-100 pH 7.6, then blocked using endogenous peroxidase with 3% hydrogen peroxide for 20 min followed by 3% normal goat serum for 30 min at room temperature prior to incubating overnight at 4 °C. The following day, slides were washed with Tris-buffered saline + Tween 20 (TBST) and incubated with secondary antibody conjugated to horseradish peroxidase. Slides were developed using diaminobenzidine and sections were counterstained with hematoxylin. Images were captured using a 20× objective on an inverted microscope (EVOS M5000, ThermoFisher, Waltham, MA, USA).

### 4.4. Tensile Testing

Hypothermic preservation and impact on ECM structure and functionality were further evaluated via tensile testing. Chorion membranes were cut into 2 cm × 7 cm rectangles, and PBS-soaked gauze was placed onto each end of the tissue. The pneumatic grips were separated to allow for approximately 1 cm of tissue within each grip of an Instron Model 3342 (Instron, Norwood, MA, USA). Using a 50 N load cell, force was applied to the tissue until failure and the maximum force and displacement were determined. Unprocessed and hypothermically stored chorion membranes from three donors were tested with at least *n* = 5 per donor.

### 4.5. In Vitro Simulated Wound Fluid Assay

To model a wound environment in vitro, 2 cm^2^ samples of unprocessed and hypothermically stored chorion membranes were exposed to simulated wound fluid (SWF) for up to 17 days at 37 °C with gentle rocking. SWF was prepared as previously described [39]. Every 2–3 days, the solution was replaced with fresh SWF.

At each time point, samples were collected, rinsed with deionized water to remove residual SWF, and dried at 65 °C to determine the dry weights (*n* = 3 per donor). Degradation was evaluated by utilizing dry weights and calculating the percentage of tissue remaining based on initial dry weights. Qualitatively, the structure of was evaluated using SEM as detailed above.

### 4.6. Proteomic Analysis of Key Regulatory Proteins

In order to characterize the overall composition and retention of key regulatory proteins in hypothermically stored chorion membranes, three grafts each from three independent donors of HSCM were assessed using a Human Cytokine Antibody Array Q1000 (RayBiotech, Norcross, GA, USA). HSCM was minced, homogenized, and extracted in total protein extraction buffer (ThermoFisher, Waltham, MA, USA) with a protease inhibitor cocktail (ThermoFisher, Waltham, MA, USA) overnight at 4 °C with gentle agitation. Following overnight incubation, the supernatant was removed, centrifuged, and loaded into the microarray and run per the manufacturer’s instructions. Slides were imaged and analyzed to calculate average protein content using a GenePix 4000B microarray scanner and GenePix Pro 7 software, respectively (Molecular Devices, Sunnyvale, CA, USA). Targets present at concentrations greater than 10 pg/mL were graphed to show relative contribution to the overall protein content measured. 

Following quantification, proteins detected in HSCM were annotated with their associated Gene Ontology annotations (The Gene Ontology Consortium 2000, The Gene Ontology Consortium 2020) using UniProtKB (The UniProt Consortium 2021), collated to determine the most abundant molecular functions, and represented by the identified proteins. Of note, proteins may be tagged with more than one term describing molecular function.

### 4.7. In Vitro MMP Inhibition Assay

HSCM was further evaluated in vitro using a collagenase inhibition assay, EnzCheck Gelatinase/Collagenase Assay (Invitrogen, Waltham, MA, USA). Then, 6 mm biopsy punches from hypothermically stored chorion membranes (*n* = 3 from 3 independent donors) were incubated for 30 min with 0.4 U/mL collagenase type IV (included in kit). Dye-quenched gelatin was added to the supernatants from these reactions and fluorescence measured after 30 min. Results were compared to a negative (no inhibition) control (collagenase IV) to determine reduction in MMP activity.

### 4.8. In Vitro Cell Attachment

Primary human fibroblast attachment and proliferation on HSCM was evaluated in vitro. Two independent lots of primary human fibroblasts (Lonza Bioscience, Morrisville, NC, USA) were thawed and cultured per manufacturer instructions. HSCM (*n* = 1 donor) was cut using a 12 mm biopsy punch and placed into microcentrifuge tubes with fibroblast cell suspension of 150,000 cells/1.5 mL per tube and placed on an orbital rotator at 37 °C and 5% CO_2_ for two hours to allow for initial cell attachment. After attachment, seeded scaffolds were transferred to an ultra-low attachment 24-well plate with 1 mL of growth media (GM, Dulbecco’s modified Eagle’s medium supplemented with 10% fetal bovine serum). On the day following initial seeding, grafts were moved to a fresh ultra-low attachment plate in 1 mL of GM. On days 1, 3, and 7, cell attachment was evaluated on 3 grafts per cell lot via AlamarBlue (ThermoFisher, Waltham, MA, USA). AlamarBlue solution was added to wells and incubated for 3 h. Percent reduction in AlamarBlue was calculated, and cell numbers were normalized by subtracting out the values for non-seeded HSCM to account for the metabolic activity of HSCM. Grafts at all timepoints were fixed in 4% paraformaldehyde for a minimum of 24 h and stained with standard H&E techniques as described above for qualitative assessment of cell attachment and infiltration.

### 4.9. Statistical Analysis

All statistical analysis was completed using GraphPad Prism (GraphPad Software, Boston, MA, USA). A one-way analysis of variance (ANOVA) with Tukey’s post hoc test was conducted to assess statistical differences in tissue thickness studies. To evaluate differences in maximum force and displacement, a nested *t*-test was utilized. A two-way ANOVA with Tukey’s multiple comparisons was conducted to assess statistical differences in tissue weight for in vitro degradation studies. A one-phase decay fit was conducted to determine the rate of in vitro degradation. A two-way ANOVA with Sidak’s post hoc test was conducted to assess statistical differences in percent tissue remaining for in vitro degradation studies and between timepoints for cellular attachment and proliferation. Outliers were removed using the ROUT method. For all graphs, average ± standard deviation is reported.

## Data Availability

The data that support the findings of this study are available from the corresponding author upon reasonable request.

## Supplementary Materials

The following supporting information can be downloaded at https://www.mdpi.com/article/10.3390/ijms242115786/s1.
