# Supplementary material for: Retention of Key Characteristics of Unprocessed Chorion Tissue Resulting in a Robust Scaffold to Support Wound Healing
Source: Int J Mol Sci. 2023 Oct 31;24(21):15786. doi: 10.3390/ijms242115786 (PMC10649069; doi:10.3390/ijms242115786)
Supplement: Supplementary file 1 [file ijms-24-15786-s001.zip › ijms-2649989-supplementary.pdf]

actin filament binding [GO:0051015]	growth factor activity [GO:0008083]	positive regulation of cell population proliferation [GO:0008284]
antigen binding [GO:0003823]	growth factor binding [GO:0019838]	positive regulation of DNA-binding transcription factor activity [GO:0051091]
ATP binding [GO:0005524]	growth hormone receptor binding [GO:0005131]	positive regulation of myeloid cell differentiation [GO:0045639]
ATPase binding [GO:0051117]	heparin binding [GO:0008201]	positive regulation of peptidyl-serine phosphorylation [GO:0033138]
BMP receptor binding [GO:0070700]	hormone activity [GO:0005179]	positive regulation of peptidyl-tyrosine phosphorylation [GO:0050731]
cadherin binding [GO:0045296]	Hsp90 protein binding [GO:0051879]	positive regulation of phosphatidylinositol 3-kinase signaling [GO:0014068]
calcium-dependent protein kinase C activity [GO:0004698]	identical protein binding [GO:0042802]	positive regulation of protein binding [GO:0032092]
carbohydrate binding [GO:0030246]	immune response [GO:0006955]	positive regulation of protein kinase B signaling [GO:0051897]
CCR chemokine receptor binding [GO:0048020]	insulin receptor binding [GO:0005158]	positive regulation of transcription by RNA polymerase II [GO:0045944]
CCR1 chemokine receptor binding [GO:0031726]	insulin-like growth factor binding [GO:0005520]	prolactin receptor binding [GO:0005148]
CCR2 chemokine receptor binding [GO:0031727]	insulin-like growth factor I binding [GO:0031994]	protease binding [GO:0002020]
CCR4 chemokine receptor binding [GO:0031729]	insulin-like growth factor II binding [GO:0031995]	protein heterodimerization activity [GO:0046982]
CCR5 chemokine receptor binding [GO:0031730]	insulin-like growth factor receptor binding [GO:0005159]	protein homodimerization activity [GO:0042803]
CD4 receptor binding [GO:0042609]	integrin binding [GO:0005178]	protein kinase activity [GO:0004672]
cellular response to cytokine stimulus [GO:0071345]	interleukin-1 receptor antagonist activity [GO:0005152]	protein phosphatase binding [GO:0019903]
cellular response to lipopolysaccharide [GO:0071222]	interleukin-1 receptor binding [GO:0005149]	protein self-association [GO:0043621]
chemoattractant activity [GO:0042056]	interleukin-1 type I receptor antagonist activity [GO:0045352]	protein tyrosine kinase activator activity [GO:0030296]
chemokine activity [GO:0008009]	interleukin-1 type II receptor antagonist activity [GO:0045353]	protein tyrosine kinase activity [GO:0004713]
chemoattractant activity involved in axon guidance [GO:1902379]	interleukin-1, type I receptor binding [GO:0005150]	protein tyrosine phosphatase activator activity [GO:0008160]
chemokine activity [GO:0008009]	interleukin-1, type II receptor binding [GO:0005151]	protein-containing complex binding [GO:0044877]
chemokine binding [GO:0019956]	interleukin-12 alpha subunit binding [GO:0042164]	receptor ligand activity [GO:0048018]
chemokine receptor antagonist activity [GO:0046817]	interleukin-12 receptor binding [GO:0005143]	receptor signaling protein tyrosine kinase activator activity [GO:0030298]
chemokine receptor binding [GO:0042379]	interleukin-2 receptor binding [GO:0005134]	receptor tyrosine kinase binding [GO:0030971]
chromatin binding [GO:0003682]	interleukin-5 receptor binding [GO:0005137]	receptor-receptor interaction [GO:0090722]

collagen binding [GO:0005518]	interleukin-6 receptor binding [GO:0005138]	regulation of actin filament organization [GO:0110053]
copper ion binding [GO:0005507]	interleukin-7 receptor binding [GO:0005139]	response to ethanol [GO:0045471]
coreceptor activity [GO:0015026]	interleukin-8 receptor binding [GO:0005153]	signaling receptor activity [GO:0038023]
co-receptor binding [GO:0039706]	kappa-type opioid receptor binding [GO:0031851]	signaling receptor binding [GO:0005102]
CXCR chemokine receptor binding [GO:0045236]	kinase activator activity [GO:0019209]	superoxide-generating NADPH oxidase activator activity [GO:0016176]
CXCR3 chemokine receptor binding [GO:0048248]	kinase activity [GO:0016301]	transcription cis-regulatory region binding [GO:0000976]
cytokine activity [GO:0005125]	kinase binding [GO:0019900]	transforming growth factor beta binding [GO:0050431]
cytokine binding [GO:0019955]	macrophage colony-stimulating factor receptor activity [GO:0005011]	transmembrane receptor protein tyrosine kinase activator activity [GO:0030297]
cytokine receptor activity [GO:0004896]	macrophage colony-stimulating factor receptor binding [GO:0005157]	transmembrane receptor protein tyrosine kinase activity [GO:0004714]
cytokine-mediated signaling pathway [GO:0019221]	MAP kinase kinase kinase activity [GO:0004709]	transmembrane signaling receptor activity [GO:0004888]
death receptor agonist activity [GO:0038177]	metal ion binding [GO:0046872]	tumor necrosis factor binding [GO:0043120]
double-stranded DNA binding [GO:0003690]	metalloendopeptidase inhibitor activity [GO:0008191]	tumor necrosis factor receptor activity [GO:0005031]
enzyme binding [GO:0019899]	molecular function inhibitor activity [GO:0140678]	tumor necrosis factor receptor binding [GO:0005164]
epidermal growth factor binding [GO:0048408]	negative regulation of neuron death [GO:1901215]	type I transforming growth factor beta receptor binding [GO:0034713]
epidermal growth factor receptor binding [GO:0005154]	nerve growth factor receptor binding [GO:0005163]	type II interferon receptor binding [GO:0005133]
epidermal growth factor receptor activity [GO:0005006]	nuclear receptor coactivator activity [GO:0030374]	type II transforming growth factor beta receptor binding [GO:0005114]
fibroblast growth factor receptor binding [GO:0005104]	peptidase inhibitor activity [GO:0030414]	type III transforming growth factor beta receptor binding [GO:0034714]
fibronectin binding [GO:0001968]	phosphatidylinositol phospholipase C activity [GO:0004435]	ubiquitin protein ligase binding [GO:0031625]
glial cell-derived neurotrophic factor receptor binding [GO:0030116]	phospholipase activator activity [GO:0016004]	vascular endothelial growth factor binding [GO:0038085]
glycosphingolipid binding [GO:0043208]	platelet-derived growth factor binding [GO:0048407]	vascular endothelial growth factor receptor activity [GO:0005021]
granulocyte differentiation [GO:0030851]	platelet-derived growth factor receptor binding [GO:0005161]	virus receptor activity [GO:0001618]
granulocyte macrophage colony-stimulating factor receptor binding [GO:0005129]	positive regulation of actin filament polymerization [GO:0030838]	zinc ion binding [GO:0008270]

Table S1: Molecular functions tagged in 53 proteins detected in hypothermically stored chorion membrane.
